# Methodological aspects for accelerometer-based assessment of physical activity in heart failure and health

**DOI:** 10.1186/s12874-021-01350-6

**Published:** 2021-11-14

**Authors:** Fabian Schwendinger, Jonathan Wagner, Denis Infanger, Arno Schmidt-Trucksäss, Raphael Knaier

**Affiliations:** 1 Division of Sports and Exercise Medicine, Department of Sport, Exercise and Health, University of Basel, Grosse Allee 6, 4052 Basel, Switzerland; 2 Division of Sleep Medicine, Harvard Medical School, Boston, MA USA; 3 Medical Chronobiology Program, Division of Sleep and Circadian Disorders, Departments of Medicine and Neurology, Brigham and Women’s Hospital, Boston, MA USA

**Keywords:** Activity monitor, Sedentary behavior, Leisure activities, Wearing time

## Abstract

**Background:**

For valid accelerometer-assessed physical activity (PA) data, several methodological aspects should be considered. We aimed to 1) visualize the applicability of absolute accelerometer cut-offs to classify PA intensity, 2) verify recommendations to measure PA over 7 days by examining inter-day variability and reactivity, 3) examine seasonal differences in PA, and 4) recommend during which 10 h day period accelerometers should be worn to capture the most PA in patients with heart failure (HEART) and healthy individuals (HEALTH).

**Methods:**

Fifty-six HEART (23% female; mean age 66 ± 13 years) and 299 HEALTH (51% female; mean age 54 ± 19 years) of the COmPLETE study wore accelerometers for 14 days. Aim 1 was analyzed descriptively. Key analyses were performed using linear mixed models.

**Results:**

The results yielded poor applicability of absolute cut-offs. The day of the week significantly affected PA in both groups. PA-reactivity was not present in either group. A seasonal influence on PA was only found in HEALTH. Large inter-individual variability in PA timing was present.

**Conclusions:**

Our data indicated that absolute cut-offs foster inaccuracies in both populations. In HEART, Sunday and four other days included in the analyses seem sufficient to estimate PA and the consideration of seasonal differences and reactivity seems not necessary. For healthy individuals, both weekend days plus four other days should be integrated into the analyses and seasonal differences should be considered. Due to substantial inter-individual variability in PA timing, accelerometers should be worn throughout waking time. These findings may improve future PA assessment.

**Trial registration:**

The COmPLETE study was registered at clinicaltrials.gov (NCT03986892).

**Supplementary Information:**

The online version contains supplementary material available at 10.1186/s12874-021-01350-6.

## Background

Physical activity (PA) is of major importance for healthy aging [1, 2] as well as the prevention and treatment of chronic non-communicable diseases such as chronic heart failure, diabetes, or cancer [3]. In patients with heart failure (HEART), structured PA is associated with lower rates of hospital readmission and cardiac mortality [4]. To investigate the dose–response relationship between PA and various health outcomes, it is central to assess PA with sufficient rigor [5, 6]. Accelerometers allow an objective measurement of PA and have been used for decades in many large-scale cohort studies such as the Generation 100 study [7], the 2005–2006 National Health and Nutrition Examination Survey [8], or the UK Biobank study [9]. The methodology for using accelerometers has evolved, intending to get the most representative picture of a population’s real-life PA [6].

When assessing PA, many methodological decisions need to be made regarding data processing as well as data collection. Such aspects include for example which cut-off values to use to determine different categories of PA intensities or for how many days subjects will be monitored. Bearing in mind that as little as 15 min of moderate PA per day reduce all-cause mortality and increase life expectancy [5, 10], small inaccuracies in the assessment of PA can lead to major errors in the conclusions. This work addresses one methodological question regarding data processing and three questions regarding data collection.

Firstly, cut-off values are used to enable the classification of performed activities into light, moderate, or vigorous intensity of PA (LPA, MPA, VPA) and are usually linked to more coherent units such as metabolic equivalents (METs) [11]. However, the applicability of this original approach is limited. Research using the ActiGraph accelerometer comparing groups with different fitness levels has shown that despite exercising at the same relative intensity, significantly different acceleration was measured [12]. The current study will extend these findings by illustrating how this issue might affect PA classification accuracy by reference to patients with heart failure and a large healthy population providing a wide cardiorespiratory fitness spectrum. Oxygen uptake in absolute terms and as a percentage of the subject’s peak oxygen uptake ($\overset{.}{\mathrm{V}}\mathrm{O}_{2\text{peak}}$), is the most suitable representative for PA intensity in this context [2, 13]. It directly reflects energy expenditure and has been used in numerous validation studies to develop the available cut-off values [11, 12].

Secondly, to obtain a representative picture of real-life PA patterns, the most recent recommendation for healthy individuals suggests the inclusion of at least four weekdays and one weekend day [14]. Hence, it is recommended to monitor PA over 7 consecutive days [14]. Such recommendations are commonly based on the fact that PA levels differ between weekday and weekend days with less PA on Sunday compared to all other days [14, 15]. To date, it is unclear if these differences in PA between weekdays and weekends are also present in patients with heart failure and adults across a wide age spectrum. A further factor to consider when defining the required monitoring duration is reactivity. Reactivity implies that at the beginning of an observation period, people that are aware of them being monitored, alter their behavior [6, 16, 17]. This has been examined in children [18], adolescents [18], middle-aged adults [17], and elderly people [16] with the results being inconclusive. However, data in patients with heart failure is missing and the majority of studies assessed reactivity only over a period of seven days [16, 18].

Thirdly, the season of the year (i.e., summer vs. winter) and the inherent weather conditions (i.e., temperature, wind, precipitation, daylight) have been shown to affect PA levels in a variety of age groups of the general population using numerous self-reported and objective tools [16, 19]. Again, there is currently no objective information available on whether this also applies to patients with heart failure and this has not been investigated over a broad age spectrum in healthy subjects.

Fourthly, when PA is measured in research, the term wear time compliance is used to describe how long the devices were worn by the subjects. Since it is difficult to achieve 100% wear time compliance, it was generally accepted over the last decade that a minimum wear time of 10 h per day provides a valid estimate of daily PA. Although the current consensus is that all data exceeding this threshold are included in the statistical analyses [7, 8, 20], there is debate whether this duration is sufficient [21]. This standard was recently compared to a wear time of 14 h per day [21]. The results indicated an error ranging from 28.2% to 41.6% (sedentary to vigorous PA) for 10 h per day, showing 14 h per day to more accurately reflect daily PA [21]. While it is not surprising that extended wear time leads to higher PA levels, it still raises the question of how to achieve such a high wear time compliance of 14 h per day. Hence, if the subjects are only willing to wear the device for shorter periods, the obvious goal needs to be to provide subjects with instructions during which 10 h of the day (i.e., the most active hours) they are supposed to wear the device.

Accordingly, the aims of this study were 1) to illustrate the PA classification accuracy of current absolute accelerometer cut-offs in relation to cardiorespiratory fitness 2) to assess differences between weekdays and weekend days as well as reactivity over a period of 14 days, 3) to examine the influence of the season of the year on PA patterns, and 4) to determine during which 10 h period during the day subjects are the most active.

## Methods

### Study design

The COmPLETE study is a cross-sectional study conducted between January 2018 and December 2019 at the Department of Sport, Exercise and Health at the University of Basel, Switzerland and is registered at clinicaltrials.gov (NCT03986892). The study aimed to perform a comprehensive assessment of components of physical fitness and cardiovascular function in HEART and healthy individuals (HEALTH) as well as to identify the most important factors contributing to healthy aging [22]. More details on the study design were reported elsewhere [22]. The protocol was approved by the Ethics Committee of North-western and Central Switzerland (EKNZ 2017–01,451) and all procedures followed the Declaration of Helsinki. Signed informed consent was obtained by all participants before study onset.

### Study participants

This study includes both, HEART (*n* = 56) and HEALTH (*n* = 299). A full description of the recruitment procedures can be found elsewhere [22]. To be eligible for HEART, participants had to be between 20–100 years of age and diagnosed with stable chronic heart failure according to the European Society of Cardiology guidelines for the diagnosis and treatment of acute and chronic heart failure [23]. To be eligible for HEALTH, participants had to be healthy, non-smoking or with no history of smoking within the last 10 years, body mass index < 30 kg^.^m^−2^, and aged between 20 to 100 years. Exclusion criteria were: manifested exercise limiting chronic disease (e.g., myocardial infarction; stroke; heart failure; lower-extremity artery disease; cancer with general symptoms; diabetes; clinically apparent renal failure; severe liver disease; chronic bronchitis GOLD stages II to IV; osteoporosis), pregnancy or breastfeeding, drug or alcohol abuse, hypertonic blood pressure > 160/100 mmHg, compromising orthopedic problems, Alzheimer’s disease or any other form of dementia, and inability to follow the procedures of the study [22].

### Participant Screening and General Health Assessment

Before the first visit, health- and smoking status, as well as physical activity readiness, were assessed via telephone interview [22]. On-site, height was measured and body composition was evaluated using a four-segment bioelectrical impedance analysis (InBody 720, InBody Co Ltd, Seoul, South Korea). In the further course of the testing procedures, blood samples were drawn via venipuncture by trained medical staff [24]. Among other blood parameters, n-terminal pro b-type natriuretic peptide concentrations were measured using a chemiluminescent microparticle immunoassay (Architect, Abbott, IL, United States) as a blood marker indicating heart failure [24].

### PA measurement

PA was objectively assessed using the GENEActiv triaxial accelerometer (Activinsights Ltd., Kimbolton, UK) [11]. The participants were asked to wear the device on their non-dominant wrist [25], 24 h per day for 14 consecutive days in their free-living conditions. PA surveillance started at midnight, the day after the participants received the device. The sampling frequency was set to 50 Hz. The collected raw data were exported using the GENEActiv software version 2.9 (Activinsights Ltd., Kimbolton, UK) and stored in binary format. All further data processing and data analyzes were done using the R-package GGIR version 2.1–3 in R (R Foundation for Statistical Computing, Vienna, Austria) [26]. As part of this, auto-calibration using local gravity as a reference and the sleep detection function were applied [27, 28]. Non-wear time was estimated based on the standard deviation and value range of the raw accelerometer data from each axis using 60 min windows [29]. The magnitude of dynamic acceleration was calculated as the vector magnitude of x-, y-, and z-axes averaged over 5-s epochs [14] and corrected for gravity with negative values rounded to zero, yielding Euclidean Norm Minus One (ENMO) in gravitational units (*g*) (1) [30].

1 $$ENMO \left(x, y, z\right)=\sqrt{{x}^{2}+{y}^{2}+{z}^{2}}-1$$

For a reliable assessment of LPA, MPA, and VPA, only subjects with wear time ≥ 10 h per day [8, 20] and valid data of at least four weekdays and one weekend day for each of the two weeks were included in the statistical analyses [14, 15]. The rationale for these rather strict criteria was to accurately assess PA patterns in both weeks, as well as during the week and on weekend days. To categorize the measured acceleration into PA intensity zones 0.03, 0.1, and 0.4 g were used as cut-offs for LPA (≥ 2 METs), MPA (≥ 3 METs), and VPA (≥ 6 METs), respectively [30, 31]. Total physical activity (TPA) was calculated by summarizing LPA, MPA, and VPA. Every activity below 0.03 g was categorized as sedentary time [30, 31].

### Cardiopulmonary exercise testing

Cardiopulmonary exercise testing was performed on a magnetically braked bicycle ergometer (Ergoselect 200; ergoline GmbH, Bitz, Germany) following one of five ramp protocols described in detail elsewhere [23]. The applied exhaustion criteria can be found in Wagner et al. [32]. Parameters of ventilation and gas exchange were collected breath-by-breath and analyzed in 10 s intervals using the MetaMax 3B portable metabolic system (Cortex Biophysik GmbH, Leipzig, Germany) [33]. Peak oxygen uptake ($\overset{.}{\mathrm{V}}\mathrm{O}_{2\text{peak}}$) was reported as the three highest consecutive V̇O_2_-values at any point during the test (30 s mean).

### Statistical analysis

All statistical analyses were performed in R. Figures were made with GraphPad Prism version 9.0.2. Data in text and tables are presented as mean ± *SD* unless stated otherwise. Figures are shown as mean ± *SE*. For Fig. 2, absolute intensities of 3 METs and 6 METs were chosen corresponding to the cut-offs for MPA and VPA, respectively according to Garber et al. [2]. Since the R-package GGIR merely includes data that at least have a midnight timestamp, only 13 days were available for analyses. To explore the impact of the weekdays vs. weekend days and week 1 vs. week 2 on PA patterns, linear mixed models were used. Weekend days were defined as Saturday and Sunday. To explore the impact of individual days of the week (i.e., Monday to Sunday) and the number of the measurement day (day 1 to 13) on PA patterns, again, linear mixed models were used. Exploratively, moderate-to-vigorous PA (MVPA) accumulated as ≥ 10 consecutive minutes was analyzed in the same way. Similarly, seasonal differences in PA patterns were investigated via linear mixed models. Seasons of the year were defined as Spring (March, April, and May), Summer (June, July, and August), Autumn (September, October, and November), and Winter (December, January, and February). Weighted linear mixed models were used to correct for heteroscedasticity, where applicable. All these models were done for LPA, MPA, MVPA, VPA, and TPA, respectively. The same methods were applied to wear time analyses. All models were adjusted for age, sex, and $\overset{.}{\mathrm{V}}\mathrm{O}_{2\text{peak}}$. Models analyzing PA, additionally included daily wear time. Differences in sleeping time between weekdays and weekend days were assessed using linear regression analyses. Differences in mean wear time between HEART and HEALTH were analyzed by Wilcoxon rank-sum test. The level of statistical significance was set to *P* = 0.05 for two-sided tests.

## Results

### Study Participants

**Fig. 1:**
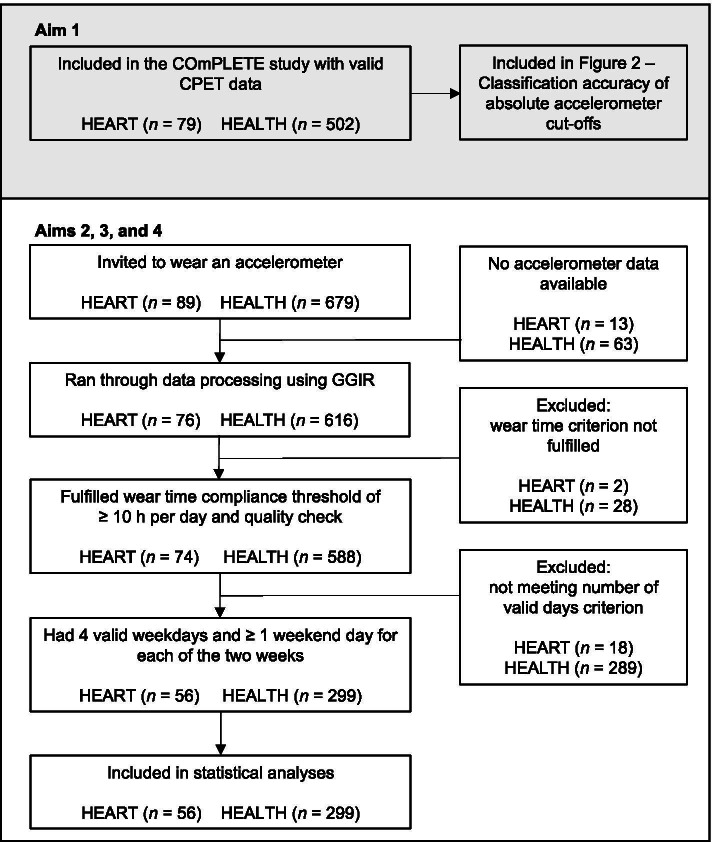
Participant flow. GGIR is the R-package used for data processing [25]. Abbreviations: HEART, patients with heart failure; HEALTH, healthy individuals; CPET, cardiopulmonary exercise testing

The flowchart is visualized in Fig. 1 and subject characteristics are displayed in Table 1 for HEART and HEALTH separately. Out of 962 and 7644 possible days, 89.6% and 92.3% fulfilled the wear time compliance threshold of ≥ 10 h and the quality check in HEART and HEALTH, respectively. After excluding subjects not fulfilling these criteria, 95.1% and 96.0% of 728 and 3887 possible days were included in the statistical analyses for HEART and HEALTH, respectively.

**Table 1:** Subjects characteristics grouped by patients with heart failure and healthy individuals

Variable	HEART (*n* = 56)	HEALTH (*n* = 299)
Female sex, *n* (*%*)	13 (23)	151 (51)
Age, years (range)	66 ± 13 (26 – 89)	54 ± 19 (21 – 91)
Height, cm	172 ± 9	171 ± 9
Body mass, kg	82.7 ± 16.9	69.6 ± 9.0
Smoking status, *n* (%)		
Smokers	5 (10)	0 (0)
Never smoked	27 (53)	238 (80)
Ex-smoker > 10 years	19 (37)	61 (20)
NYHA class, *n* (%)		
I	26 (47)	0 (0)
II	18 (33)	0 (0)
III	11 (20)	0 (0)
Daily accelerometer wear time, min	1434.2 ± 44.2	1434.8 ± 42.4
$\overset{.}{\mathrm{V}}\mathrm{O}_{2\text{peak}}$, mL^.^kg^−1.^min^−1^ (range)	21.5 ± 6.4 (10.2 – 39.5)	34.4 ± 9.9 (14.2 – 65.1)
NT-proBNP pg/mL	589.0 ± 677.2	119.9 ± 259.4

Data are presented as mean ± SD unless stated otherwise. There were missing data in the heart failure group (HEART) for smoking status (*n* = 5), NYHA class (*n* = 1) and NT-proBNP (*n* = 1). Among the healthy participants (HEALTH), data was missing for NT-proBNP (n = 1). *Abbreviations*: *NYHA class* New York Association functional classification, $\overset{.}{\mathrm{V}}\mathrm{O}_{2\text{peak}}$ Peak oxygen uptake, *NT-proBNP* N-terminal pro b-type natriuretic peptide, *SD* Standard deviation.

### Accuracy of current cut-offs in the classification of PA intensities

**Fig. 2:**
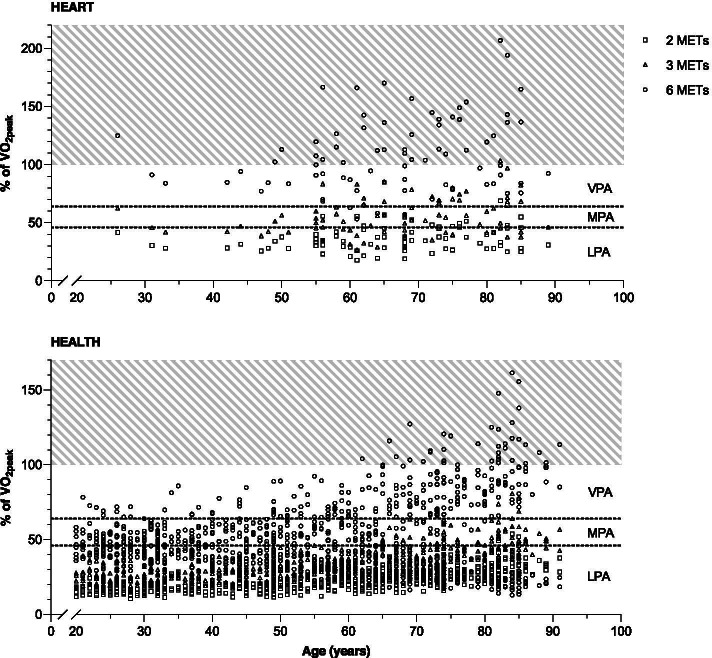
Cardiorespiratory response in % of $\overset{.}{\mathrm{V}}\mathrm{O}_{2\text{peak}}$ to exercising at 2, 3, and 6 METs across the age spectrum (20 to 91 years) for patients with heart failure (HEART) and healthy individuals (HEALTH). The age of all participants is displayed on the x-axes. The relative intensity in % of the subjects’ $\overset{.}{\mathrm{V}}\mathrm{O}_{2\text{peak}}$ that would be required to exercise at the three absolute intensities (2, 3, and 6 METs) is depicted on the y-axes. Intensity categories are marked by the dashed lines. LPA is defined as 0 to < 46% of $\overset{.}{\mathrm{V}}\mathrm{O}_{2\text{peak}}$, MPA as 46 to < 64% of $\overset{.}{\mathrm{V}}\mathrm{O}_{2\text{peak}}$, and VPA as 64 to 100% of $\overset{.}{\mathrm{V}}\mathrm{O}_{2\text{peak}}$.^2^ The hatched area symbolized the intensity that cannot be maintained for a prolonged time, as it exceeds the individual’s cardiorespiratory fitness. Abbreviations: LPA, light physical activity; MPA, moderate physical activity; VPA, vigorous physical activity; $\overset{.}{\mathrm{V}}\mathrm{O}_{2\text{peak}}$, peak oxygen uptake

For 5.1% of HEART, the cut-off for VPA (6 METs) was set too low, as this absolute intensity corresponded to MPA in relative terms. Yet, for 51.9%, the cut-off value was above their $\overset{.}{\mathrm{V}}\mathrm{O}_{2\text{peak}}$. Hence, even if they exercised with maximum aerobic effort, they would still be classified as being moderately active. In contrast, for just 7.6%, this was the case in HEALTH and for 53.2%, the cut-off value for VPA (6 METs) did truly demand only moderate relative intensity. Similar patterns were apparent for the cut-off for MPA (3 METs) with the absolute intensity not demanding enough relative intensity for 38.0% and 87.8% for HEART and HEALTH, respectively, thus, attributing them more MPA than was actually performed. For 25.3% and 1.0% of HEART and HEALTH, respectively, the cut-off for MPA (3 METs) was set too high and did already demand vigorous relative intensity exercising at 4.5 METs (MPA) and 8 METs (VPA), 26.6% (3.8% below and 69.6% above the category) and 10.1% (0% below and 89.9% above the category) would be classified correctly as MPA and VPA, respectively, using the intensity categories of Garber et al. [2]. This is presented graphically in Fig. 2. Further details focusing on the $\overset{.}{\mathrm{V}}\mathrm{O}_{2\text{peak}}$ spectrum of the current cohort are provided in Additional file 1: Figure 1.

### Differences between weekdays and weekend days and reactivity

**Fig. 3:**
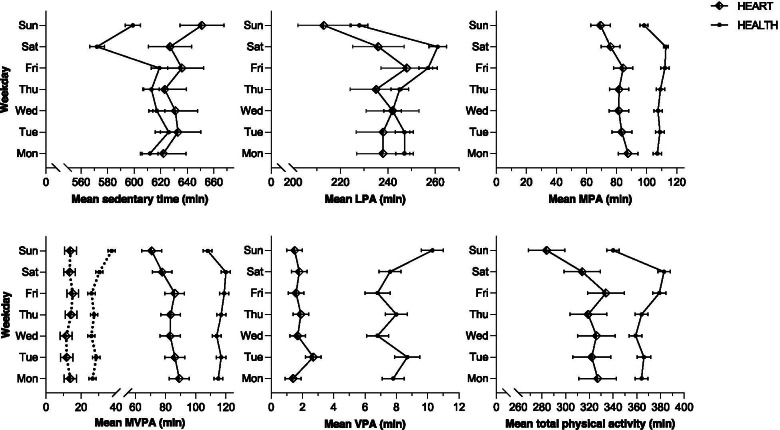
Mean physical activity patterns ± *SE* for each day of the week for patients with heart failure (HEART) and healthy individuals (HEALTH), respectively. Dotted lines in the MVPA graph additionally illustrate minutes accumulated in bouts of ≥ 10 min. Abbreviations: LPA, light physical activity; MPA, moderate physical activity; MVPA, moderate-to-vigorous physical activity; VPA, vigorous physical activity; TPA, total physical activity; *SE*, standard error

Detailed analyses of PA patterns of both HEART and HEALTH according to the day of the week are displayed in Fig. 3. In HEART, the day of the week was significantly associated with levels of TPA, MVPA, MPA, and LPA (all: *P* < 0.001), but not VPA and sedentary time. Among HEALTH, all PA intensities including sedentary time were significantly affected by this factor (all: *P* < 0.001, except VPA: *P* = 0.002). Estimates ± *SE* and 95% CI for all days and the respective PA intensities are available in Additional file 1: Table 1. MVPA in 10 min bouts was not significantly affected by the day of the week in HEART but in HEALTH (*P* < 0.001; see Fig. 3). Analyses investigating differences in PA patterns between weekdays and weekend days are presented in Additional file 1: Table 2. As a potential explanatory variable for differences in PA between the aforementioned factors, sleep time was analyzed. There was a difference in sleep time between weekdays and weekends (∆ mean = 28.4 ± 15.8 min; 95% CI: 24.1 to 32.6 min; *P* < 0.001) in HEART but not in HEALTH. There was little evidence supporting the presence of reactivity throughout the 13-day measurement period in none of the PA patterns in either group (*P* > 0.05).

### Seasonal variations in PA levels

**Fig. 4:**
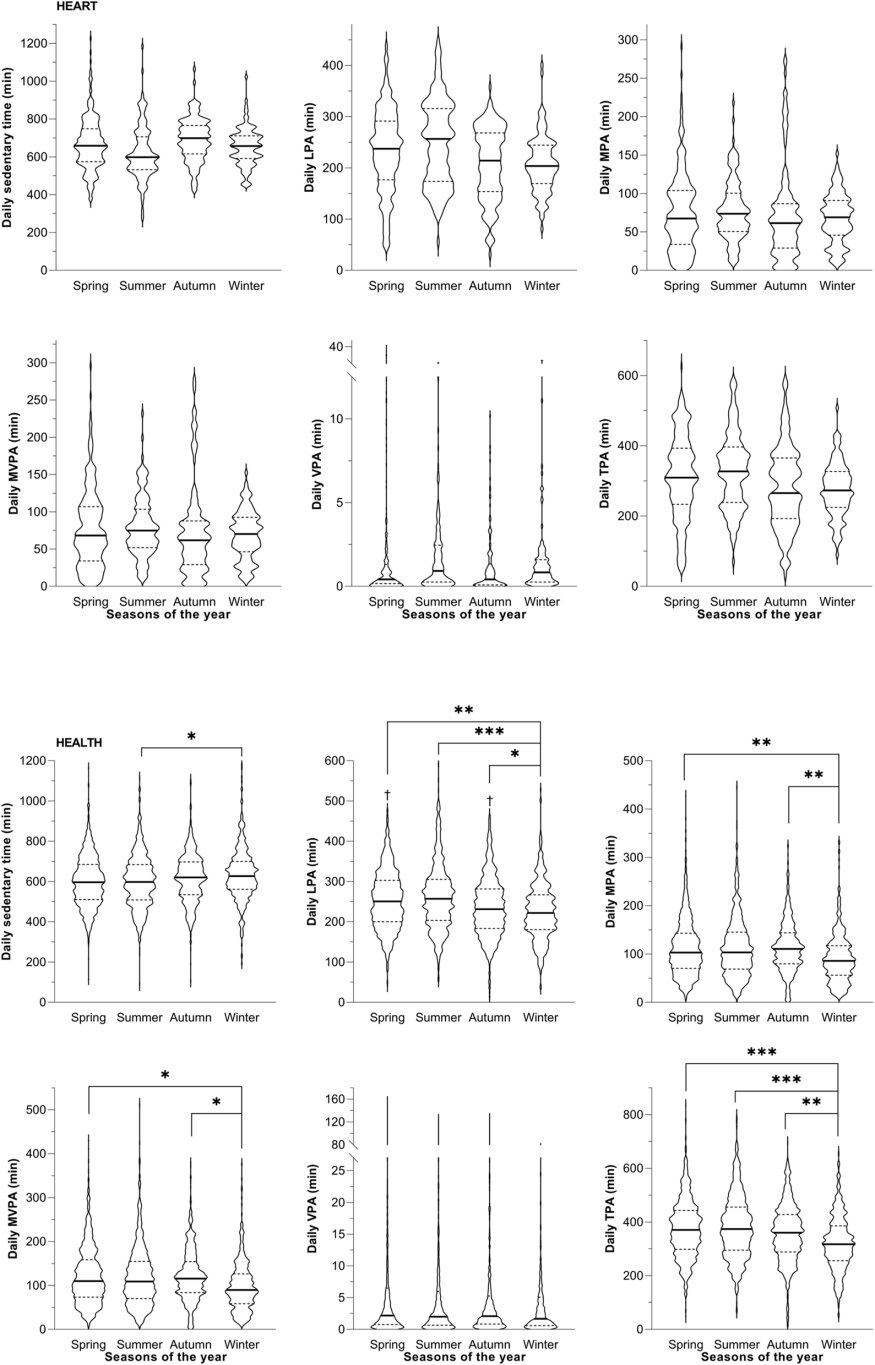
Violin plots of daily physical activity patterns of patients with heart failure (HEART) and healthy individuals (HEALTH) for each season of the year. Bold horizontal lines in the violin plots depict the median and dotted lines represent 25^th^ and 75^th^ percentiles. Number of days included in the analyses for each season of the year for HEART and HEALTH, respectively: Spring (*n* = 269 & 1589), Summer (*n* = 157 & 813), Autumn (n = 151 & 803), and Winter (*n* = 115 & 540). Abbreviations: LPA, light physical activity; MPA, moderate physical activity; MVPA, moderate-to-vigorous physical activity; VPA, vigorous physical activity; TPA, total physical activity. * *P* < .05; ** *P* < .01; *** *P* < .001; † sig. different from summer with *P* < .05

In HEART, there were no seasonal differences neither for PA patterns nor for sedentary time (see Fig. 4). However, for HEALTH, there were significant differences in TPA (*P* < 0.001), MVPA (*P* = 0.011), MPA (*P* = 0.006), LPA (*P* < 0.001), and sedentary time (*P* = 0.012) but not for VPA (see Fig. 4).

### Influence of wear time compliance on PA assessment

The measurement period had no impact on wear time compliance in HEART. Yet, for HEALTH, a significant but non-relevant reduction in wear time compliance for each progressing measurement day (-0.5 min, *SE* = 0.1 min; 95% CI: -0.8 to -0.3 min; *P* < 0.001) was apparent.

**Fig. 5:**
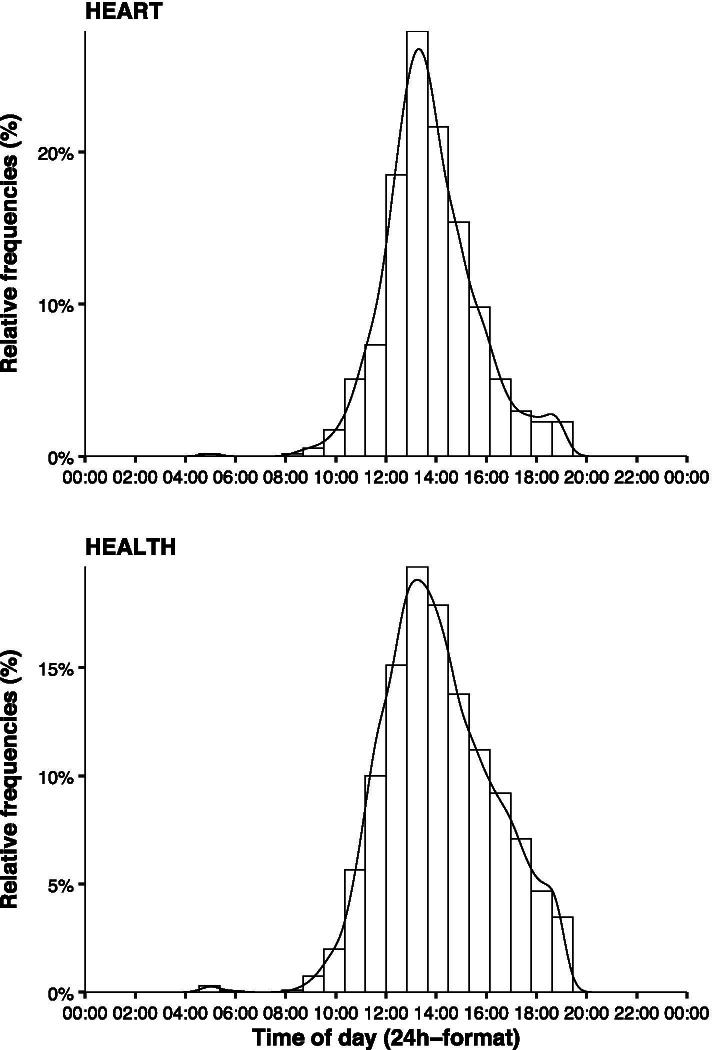
Histogram showing the distribution of the midpoint of the most active 10 h period during the day for patients with heart failure (HEART) and healthy individuals (HEALTH), respectively

The distribution of the midpoints of the most active 10 h period during the day for HEART and HEALTH are displayed in Fig. 5.

## Discussion

The major novel findings of this study were that, firstly, the use of absolute accelerometer cut-offs is accompanied by a large proportion of individuals’ PA being falsely classified. Secondly, the inclusion of Sunday and four other days may be required to estimate PA patterns in patients with heart failure while for healthy adults the inclusion of Saturday and Sunday plus four other days [15] seems necessary. Thirdly, the season of the year seems not to affect PA patterns in patients with heart failure but in healthy individuals. Fourthly, recommendations regarding the most active 10 h period cannot be made due to large inter-individual variability. Accelerometers should ideally be worn throughout waking time.

### Accuracy of current cut-offs in the classification of PA intensity

In the past, limited applicability has been shown of absolute accelerometer cut-offs in populations other than those the cut-offs were derived from [12]. This study adds details regarding the classification accuracy of such cut-offs to the original critique by Ozemek et al. [12] by analyzing a population with a wide range of cardiorespiratory fitness. The majority of all data points in Fig. 2 did not fall into the correct intensity category. The relative intensity of deconditioned individuals such as patients with heart failure or the elderly would often be far beyond the target intensity of the absolute cut-offs and vice versa for younger and/or well-trained individuals. A recent study using relative PA intensities found VPA and MVPA to lead to significant reductions in all-cause mortality [34]. Using absolute accelerometer cut-offs, it may not be possible to objectively assess this relationship because of the mismatch of absolute and relative PA intensity. Consequently, inaccurate PA data constitutes a major issue. Together with previous findings [12], this study shows that there is a need to adjust accelerometer cut-offs to the subject’s maximum oxygen uptake ($\overset{.}{\mathrm{V}}\mathrm{O}_{2\text{max}}$), particularly in the aforementioned populations. $\overset{.}{\mathrm{V}}\mathrm{O}_{2\text{max}}$-adjusted cut-offs would greatly enhance PA assessment.

### Differences between weekdays and weekend days and reactivity

Whether an accurate picture of daily PA patterns in a population of interest is obtained, is closely dependent on the number of valid days necessary for inclusion in the analyses [14, 15]. This should be determined before data collection. Dillon et al. [15] suggested that six days including both Saturday and Sunday are necessary for healthy middle-aged adults to obtain reliable estimates of weekly PA patterns. However, a more recent study in healthy young adults found five days with at least one weekend day to be sufficient [14]. The recommendations to include one or both weekend days are derived from data showing less PA on weekends compared to weekdays and more specifically, less PA on Sundays compared to all other days [14–16]. Results of the current study are in line with this.

However, distinguishing only between weekdays and weekend days may return a misleading picture. If weekend days are analyzed individually, it seems that Saturday may not be different from the other weekdays in patients with heart failure, but healthy individuals may perform relatively more LPA and sit less (thus more TPA) on this day compared to all other days, except Friday. Consequently, for patients with heart failure, the inclusion of Sunday but not Saturday may be necessary to obtain a reliable picture of the population’s PA. Combining the recommendations of Dillon et al. [15] for healthy adults with our findings, PA assessment in patients with heart failure should aim to include Sunday and five other days of the week into the statistical analyses to obtain an accurate picture of all PA patterns. For healthy individuals, our results support the recommendations of Dillon et al. [15] to include PA data of both Saturday and Sunday plus four other days.

The discrepancies regarding PA behavior on Saturday between healthy individuals in this study and the data of Ricardo et al. [14] suggest that the country in which the research is performed should be considered when setting criteria for PA assessment in future studies. Depending on the country, different days of the week may be considered days off (e.g., Sunday in Brazil, Friday in Islamic countries). Days off may leave people more time for leisure activities, leading to different PA behavior. Even though this is not covered by the present data, it seems important for countries with differing weekend definitions to include respective days off into the analyses.

Exploratively, we analyzed MVPA when only minutes accumulated in ≥ 10 min bouts were considered to see how structured activities/sports change. When comparing single minute counts with ≥ 10 min bouts (see Fig. 3) no different PA behavior in patients with heart failure was found. Yet, in healthy individuals, a decrease in activity on Sunday when every minute is considered but an increase when the latter method was used was apparent. This suggests, individuals perform more structured activities on this day but less overall PA. The longer time spent at moderate-to-vigorous intensity translates to more time at a higher heart rate and consequently greater cardiac output [35]. This in turn is an effective stimulus for the circulation and thus increased $\overset{.}{\mathrm{V}}\mathrm{O}_{2\text{max}}$. It, therefore, seems important to include both methods of measuring MVPA in PA assessment studies.

Finally, there was little evidence for reactivity in any of the PA intensities or sedentary behavior neither for patients with heart failure nor for healthy individuals. For studies that aim to obtain a representative picture of the general population’s PA, it seems appropriate to use the first 7 days the device is worn. No adjustment for reactivity seems necessary in both populations.

### Seasonal variations in PA levels

Seasonal differences in objectively measured PA are commonly described in healthy individuals with less activity in winter compared to summer [36]. Klompstra et al. [37] reported similar data for patients with heart failure obtained using a questionnaire. While in the current study there was no evidence for a seasonal effect on any of the researched PA patterns in patients with heart failure, this was true for healthy individuals. One reason for the contrasting findings could be the cross-sectional design in the current study, while Klompsta et al. [37] assessed PA in the same patients, once in summer and once in winter. The rather small sample size in our study might have been another issue. This finding needs to be confirmed in larger longitudinal studies in patients with heart failure.

### Influence of wear time compliance on PA assessment

This study provides little evidence that wear time compliance is affected to a relevant extend by the duration of the measurement period. Regarding the significant association of wear time with the measurement day, estimates were only minor and de facto not relevant.

Finally, Fig. 5 illustrates the large differences in PA between different times of the day. Furthermore, it highlights substantial inter-individual variations in PA during the day. Hence, it does not seem reasonable to recommend a certain period within a day but rather to wear the device throughout waking time, aiming to achieve at least 13 h of wear time per day as previously recommended [21].

### Limitations

Limitations of this study were, firstly, that no accelerometer data were available during the cardiorespiratory exercise testing since it was performed on a bicycle ergometer, thus only allowing a theoretical calculation of classification accuracy of absolute cut-offs. Secondly, the strict criteria used to define a valid day may have led to the inclusion of particularly compliant participants. On the other hand, these criteria may have yielded accurate PA estimates for both weeks. Thirdly, the cross-sectional study design may have limited the validity of the analyses regarding seasonal differences. Fourthly, Fig. 5 shows the midpoint of the most active 10 h period for TPA. Distributions of the respective PA patterns might differ from that. Yet, it should still be recommended to wear the accelerometer throughout waking hours to capture all PA patterns. Finally, even though previous studies cast doubt on the ability of wrist-worn accelerometers to accurately measure PA [13], a recent study comparing the energy expenditure assessed using wrist-worn accelerometry to doubly-labeled water found wrist-worn accelerometers to be a precise tool for estimating energy expenditure in the free-living general population [38]. Nonetheless, data collected at different wear locations should be compared with caution [39].

## Conclusion

To conclude, absolute cut-offs constitute a source of error for PA classification and $\overset{.}{\mathrm{V}}\mathrm{O}_{2\text{max}}$-adjusted cut-offs would greatly enhance PA assessment. Furthermore, Sunday and any five other days [14] should be included in the statistical analyses to obtain a reliable picture of all PA patterns in patients with heart failure. For healthy individuals, our results support the recommendation of including six days comprising Saturday and Sunday [14]. Seasonal influences on PA patterns do not seem to be present in patients with heart failure but in healthy populations. When using sealed wrist-worn accelerometers, no adjustment for reactivity seems necessary. Lastly, large inter-individual variability makes it difficult to recommend wearing the device within a fixed 10 h period during a day. In studies where 24 h per day of wear time is not possible, subjects should be encouraged to wear the device throughout waking time. The aforementioned findings may contribute to improving study design, data collection and data processing of future studies, thereby yielding a more reliable picture of real-life PA both in patients with heart failure and healthy adults.

## Supplementary Information

**Additional file 1: Table 1**. Linear mixed model results with estimates of daily physical activity (min) ± SE for patients with heart failure and healthy individuals. **Table 2**. Linear mixed model results with estimates of daily physical activity (min) ± SE for patients with heart failure and healthy individuals. **Figure 1**. Cardiorespiratory response in % of $\overset{.}{\mathrm{V}}\mathrm{O}_{2\text{peak}}$ to exercising at 2, 3, and 6 METs across the cardiorespiratory fitness spectrum ($\overset{.}{\mathrm{V}}\mathrm{O}_{2\text{peak}}$ range = 10.2 – 65.1 mL.kg-1.min-1) including patients with heart failure (HEART) and healthy individuals (HEALTH). The $\overset{.}{\mathrm{V}}\mathrm{O}_{2\text{peak}}$ of all participants is displayed on the x-axis. The relative intensity in % of the subjects’ $\overset{.}{\mathrm{V}}\mathrm{O}_{2\text{peak}}$ that would be required to exercise at the three absolute intensities (2, 3, and 6 METs) is depicted on the y-axis. Intensity categories are marked by the dashed lines. LPA is defined as 0 to < 46% of $\overset{.}{\mathrm{V}}\mathrm{O}_{2\text{peak}}$, MPA as 46 to < 64% of $\overset{.}{\mathrm{V}}\mathrm{O}_{2\text{peak}}$, and VPA as 64 to 100% of $\overset{.}{\mathrm{V}}\mathrm{O}_{2\text{peak}}$. 2 The hatched area symbolizes the intensity that cannot be maintained for a prolonged time, as it exceeds the individual’s cardiorespiratory fitness. Abbreviations: LPA, light physical activity; MPA, moderate physical activity; VPA, vigorous physical activity; $\overset{.}{\mathrm{V}}\mathrm{O}_{2\text{peak}}$, peak oxygen uptake.

## Data Availability

The data sets used and analyzed during the current study are available from the corresponding author on reasonable request.

## Abbreviations

PA: Physical activity
HEART: Patients with heart failure
LPA: Light physical activity
MPA: Moderate physical activity
VPA: Vigorous physical activity
METs: Metabolic equivalents
HEALTH: Healthy individuals
ENMO: Euclidean Norm Minus One
TPA: Total physical activity
$\overset{.}{\mathrm{V}}\mathrm{O}_{2\text{peak}}$: Peak oxygen uptake
MVPA: Moderate-to-vigorous physical activity
$\overset{.}{\mathrm{V}}\mathrm{O}_{2\text{max}}$: Maximum oxygen uptake
