# Supplementary material for: Methodological aspects for accelerometer-based assessment of physical activity in heart failure and health
Source: BMC Med Res Methodol. 2021 Nov 14;21:251. doi: 10.1186/s12874-021-01350-6 (PMC8590791; doi:10.1186/s12874-021-01350-6)
Supplement: Supplementary file 1 — Additional file 1: Table 1. Linear mixed model results with estimates of daily physical activity (min) ± SE for patients with heart failure and healthy individuals. Table 2. Linear mixed model results with estimates of daily physical activity (min) ± SE for patients with heart failure and healthy individuals. Figure 1. Cardiorespiratory response in % of $\overset{.}{\mathrm{V}}\mathrm{O}_{2\text{peak}}$V.O2peak to exercising at 2, 3, and 6 METs across the cardiorespiratory fitness spectrum ($\overset{.}{\mathrm{V}}\mathrm{O}_{2\text{peak}}$V.O2peak range = 10.2 – 65.1 mL.kg-1.min-1) including patients with heart failure (HEART) and healthy individuals (HEALTH). The $\overset{.}{\mathrm{V}}\mathrm{O}_{2\text{peak}}$V.O2peak of all participants is displayed on the x-axis. The relative intensity in % of the subjects’ $\overset{.}{\mathrm{V}}\mathrm{O}_{2\text{peak}}$V.O2peak that would be required to exercise at the three absolute intensities (2, 3, and 6 METs) is depicted on the y-axis. Intensity categories are [file 12874_2021_1350_MOESM1_ESM.docx]

**Methodological aspects for accelerometer-based assessment of physical activity in heart failure and health**

Schwendinger F.^1^, Wagner J.^1^, Infanger D.^1^, Schmidt-Trucksäss A.^1^, & Knaier R.^1,2,3^

^1^ Division of Sports and Exercise Medicine, Department of Sport, Exercise and Health, University of Basel, Basel, Switzerland

^2^ Division of Sleep Medicine, Harvard Medical School, Boston, MA, USA.

^3^ Medical Chronobiology Program, Division of Sleep and Circadian Disorders, Departments of Medicine and Neurology, Brigham and Women's Hospital, Boston, MA, USA.

Corresponding author: Arno Schmidt-Trucksäss (AST), arno.schmidt-trucksaess@unibas.ch;
Tel: +41 (0) 61 2074741; Fax: +41 (0) 61 2074742; Department of Sport, Exercise and Health, University of Basel, Birsstrasse 320 B, 4052 Basel, Switzerland; ORCID: 0000-0002-4662-3911

Fabian Schwendinger (FS), fabian.schwendinger@unibas.ch; ORCID: 0000-0001-7795-1478

Jonathan Wagner (JW), jonathan.wagner@unibas.ch; ORCID: 0000-0002-3436-588X

Denis Infanger (DI), denis.infanger@unibas.ch; ORCID: 0000-0001-9028-7110

Raphael Knaier (RK), raphael.knaier@unibas.ch; ORCID: 0000-0002-0244-7768

| Table 1. Linear mixed model results with estimates of daily physical activity (min) ± *SE* for patients with heart failure and healthy individuals. | | | | | | | | | |  |
| --- | --- | --- | --- | --- | --- | --- | --- | --- | --- | --- |
|  | **HEART (*n* = 56)** | | | |  | **HEALTH (*n* = 299)** | | | | |
| Variable | **Estimate^†^** | | ***SE*** | **95% CI** |  | | **Estimate^†^** | ***SE*** | **95% CI** |  |
| Sedentary time | | |  |  |  | |  |  |  |  |
| Monday | 622.4 | | 17.1 | 588.1 to 656.8 |  | | 612.0 | 5.9 | 600.3 to 623.6 |  |
| Tuesday | 632.8 | | 17.3 | 598.1 to 667.6 |  | | 625.8 | 6.2 | 613.5 to 638.0 |  |
| Wednesday | 631.0 | | 16.9 | 597.0 to 665.0 |  | | 617.0 | 6.0 | 605.2 to 628.7 |  |
| Thursday | 622.8 | | 16.4 | 589.9 to 655.8 |  | | 613.0 | 5.9 | 601.5 to 624.6 |  |
| Friday | 636.1 | | 16.4 | 603.1 to 669.1 |  | | 618.8 | 6.1 | 606.8 to 630.7 |  |
| Saturday | 627.2 | | 16.3 | 594.5 to 660.0 |  | | 572.5 | 5.6 | 561.4 to 583.5 |  |
| Sunday | 650.5 | | 16.6 | 617.2 to 683.8 |  | | 599.1 | 5.8 | 587.7 to 610.5 |  |
| LPA | | |  |  |  | |  |  |  |  |
| Monday | | 238.0 | 11.2 | 215.5 to 260.4 |  | | 247.2 | 3.7 | 240.0 to 254.5 |  |
| Tuesday | | 237.8 | 11.3 | 215.3 to 260.4 |  | | 246.9 | 3.8 | 239.4 to 254.3 |  |
| Wednesday | | 242.4 | 11.1 | 220.1 to 264.7 |  | | 241.7 | 3.7 | 234.4 to 248.9 |  |
| Thursday | | 235.2 | 10.9 | 213.3 to 257.1 |  | | 244.9 | 3.6 | 237.7 to 252.1 |  |
| Friday | | 248.2 | 10.9 | 226.3 to 270.2 |  | | 257.4 | 3.8 | 250.0 to 264.9 |  |
| Saturday | | 236.0 | 10.9 | 214.2 to 257.8 |  | | 260.7 | 3.7 | 253.5 to 267.9 |  |
| Sunday | | 213.3 | 10.9 | 191.4 to 235.1 |  | | 227.9 | 3.6 | 220.9 to 235.0 |  |
| MPA | | |  |  |  | |  |  |  |  |
| Monday | | 87.6 | 6.5 | 74.5 to 100.8 |  | | 107.1 | 2.7 | 101.8 to 112.3 |  |
| Tuesday | | 83.5 | 6.6 | 70.3 to 96.7 |  | | 108.8 | 2.8 | 103.3 to 114.2 |  |
| Wednesday | | 81.5 | 6.5 | 68.4 to 94.5 |  | | 107.6 | 2.7 | 102.3 to 112.9 |  |
| Thursday | | 81.6 | 6.4 | 68.8 to 94.3 |  | | 109.2 | 2.7 | 103.9 to 114.4 |  |
| Friday | | 84.4 | 6.4 | 71.7 to 97.2 |  | | 112.2 | 2.8 | 107.6 to 117.7 |  |
| Saturday | | 76.0 | 6.3 | 63.3 to 88.7 |  | | 112.8 | 2.7 | 107.6 to 118.1 |  |
| Sunday | | 69.3 | 6.4 | 56.5 to 82.2 |  | | 98.3 | 2.6 | 93.3 to 103.4 |  |
| VPA | | |  |  |  | |  |  |  |  |
| Monday | | 1.4 | 0.5 | 0.4 to 2.5 |  | | 7.8 | 0.7 | 6.3 to 9.2 |  |
| Tuesday | | 2.7 | 0.5 | 1.6 to 3.8 |  | | 8.7 | 0.8 | 7.2 to 10.2 |  |
| Wednesday | | 1.6 | 0.5 | 0.6 to 2.7 |  | | 6.8 | 0.7 | 5.3 to 8.3 |  |
| Thursday | | 1.9 | 0.5 | 0.8 to 2.9 |  | | 8.0 | 0.7 | 6.5 to 9.4 |  |
| Friday | | 1.6 | 0.5 | 0.6 to 2.6 |  | | 6.8 | 0.8 | 5.3 to 8.3 |  |
| Saturday | | 1.8 | 0.5 | 0.8 to 2.8 |  | | 7.6 | 0.7 | 6.2 to 9.0 |  |
| Sunday | | 1.5 | 0.5 | 0.5 to 2.5 |  | | 10.3 | 0.7 | 8.9 to 11.8 |  |

**Supplementary tables**

^†^adjusted for V̇O_2peak_, sex, age, and wear time. Abbreviations: HEART, patients with heart failure; HEALTH, healthy individuals; LPA, light physical activity; MPA, moderate physical activity; V̇O_2peak_, peak oxygen uptake; *SE*, standard error.

| Table 1 continued. | | | | | | | | | |
| --- | --- | --- | --- | --- | --- | --- | --- | --- | --- |
|  | **HEART (*n* = 56)** | | | |  | **HEALTH (*n* = 299)** | | | |
| Variable | **Estimate^†^** | | ***SE*** | **95% CI** |  | | **Estimate^†^** | ***SE*** | **95% CI** |
| MVPA | | |  |  |  | |  |  |  |
| Monday | | 89.1 | 6.7 | 75.6 to 102.5 |  | | 114.5 | 2.9 | 108.7 to 120.3 |
| Tuesday | | 86.2 | 6.8 | 72.6 to 99.7 |  | | 116.9 | 3.1 | 110.9 to 122.9 |
| Wednesday | | 83.1 | 6.7 | 69.7 to 96.5 |  | | 114.4 | 3.0 | 108.6 to 120.3 |
| Thursday | | 83.4 | 6.5 | 70.3 to 96.6 |  | | 116.9 | 2.9 | 111.1 to 122.7 |
| Friday | | 86.1 | 6.5 | 73.0 to 99.2 |  | | 118.8 | 3.0 | 112.8 to 124.8 |
| Saturday | | 77.8 | 6.5 | 64.7 to 90.8 |  | | 120.3 | 2.9 | 114.5 to 126.1 |
| Sunday | | 70.8 | 6.6 | 57.7 to 84.0 |  | | 107.6 | 2.9 | 102.0 to 113.2 |
| MVPA (10 min bouts) | | |  |  |  | |  |  |  |
| Monday | | 13.9 | 3.5 | 6.9 to 20.9 |  | | 26.7 | 2.0 | 22.7 to 30.7 |
| Tuesday | 11.8 | | 3.5 | 4.7 to 18.8 |  | | 28.6 | 2.1 | 24.4 to 32.8 |
| Wednesday | 11.6 | | 3.4 | 4.7 to 18.5 |  | | 25.9 | 2.1 | 21.9 to 30.0 |
| Thursday | 14.3 | | 3.3 | 7.6 to 21.0 |  | | 27.5 | 2.0 | 23.5 to 31.4 |
| Friday | 15.3 | | 3.3 | 8.6 to 21.9 |  | | 26.0 | 2.1 | 21.9 to 30.1 |
| Saturday | 13.5 | | 3.3 | 6.8 to 20.1 |  | | 30.5 | 2.0 | 26.5 to 34.4 |
| Sunday | 13.9 | | 3.4 | 7.1 to 20.6 |  | | 37.5 | 2.0 | 33.5 to 41.4 |
| TPA |  | |  |  |  | |  |  |  |
| Monday | 326.6 | | 15.9 | 294.6 to 358.5 |  | | 364.4 | 5.3 | 353.9 to 374.9 |
| Tuesday | 322.5 | | 16.0 | 290.4 to 354.5 |  | | 366.2 | 5.5 | 355.3 to 377.2 |
| Wednesday | 325.9 | | 15.8 | 294.2 to 357.6 |  | | 358.6 | 5.4 | 348.0 to 369.2 |
| Thursday | 318.7 | | 15.5 | 287.7 to 349.8 |  | | 363.7 | 5.3 | 353.3 to 374.2 |
| Friday | 334.0 | | 15.5 | 303.9 to 365.2 |  | | 378.7 | 5.5 | 367.9 to 389.4 |
| Saturday | 313.6 | | 15.4 | 282.6 to 344.5 |  | | 383.1 | 5.3 | 372.7 to 393.5 |
| Sunday | 283.7 | | 15.4 | 252.8 to 314.7 |  | | 339.7 | 5.3 | 329.3 to 350.1 |

^†^adjusted for V̇O_2peak_, sex, age, and wear time. Abbreviations: HEART, patients with heart failure; HEALTH, healthy individuals; MVPA, moderate-to-vigorous physical activity; 10 min bouts, minutes accumulated in activity periods of ≥ 10 min; VPA, vigorous physical activity; TPA, total physical activity; V̇O_2peak_, peak oxygen uptake; *SE*, standard error.

| Table 2. Linear mixed model results with estimates of daily physical activity (min) ± *SE* for patients with heart failure and healthy individuals. | | | | | | | | | |
| --- | --- | --- | --- | --- | --- | --- | --- | --- | --- |
|  | **HEART (*n* = 56)** | | | |  | **HEALTH (*n* = 299)** | | | |
| Variables | **Estimate^†^** | ***SE*** | **95% CI** | ***P*-value** |  | **Estimate^†^** | ***SE*** | **95% CI** | ***P*-value** |
| Weekdays vs. weekend |  |  |  |  |  |  |  |  |  |
| Sedentary time | 10.4 | 7.7 | -4.7 to 25.6 | .178 |  | -31.6 | 3.7 | -38.7 to -24.4 | < .001*** |
| LPA | -15.9 | 4.0 | -23.7 to -8.1 | < .001*** |  | -2.9 | 2.1 | -6.9 to 1.2 | .169 |
| MPA | -6.6 | 1.5 | -9.5 to -3.6 | < .001*** |  | -3.2 | 1.5 | -6.1 to -0.3 | .033* |
| MVPA | -6.4 | 1.5 | -9.4 to -3.5 | < .001*** |  | -2.1 | 1.6 | -5.3 to 1.1 | .198 |
| VPA | -0.1 | 0.2 | -0.6 to 0.3 | .555 |  | 1.3 | 0.5 | 0.3 to 2.4 | .013* |
| TPA | -27.0 | 5.5 | -37.7 to -16.2 | < .001*** |  | -4.3 | 3.1 | -10.4 to 1.9 | .176 |

^†^adjusted for V̇O_2peak_, sex, age, and wear time. * *P* < .05; ** *P* < .01; *** *P* < .001. Abbreviations: HEART, patients with heart failure; HEALTH, healthy individuals; LPA, light physical activity; MPA, moderate physical activity; MVPA, moderate-to-vigorous physical activity; VPA, vigorous physical activity; TPA, total physical activity; V̇O_2peak_, peak oxygen uptake; *SE*, standard error.

**Supplementary figure**

**
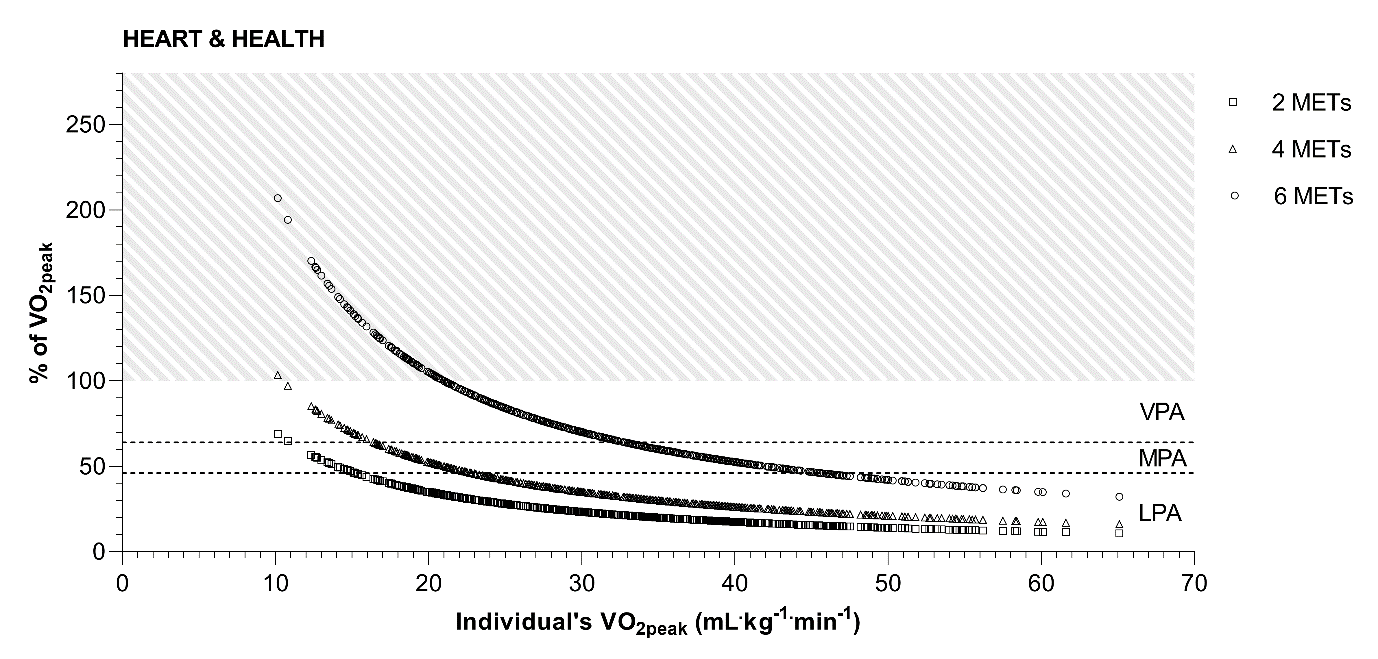
**

**Figure 1.** Cardiorespiratory response in % of V̇O_2peak_ to exercising at 2, 3, and 6 METs across the cardiorespiratory fitness spectrum (V̇O_2peak_ range = 10.2 – 65.1
mL^.^kg^-1.^min^-1^) including patients with heart failure (HEART) and healthy individuals (HEALTH). The V̇O_2peak_ of all participants is displayed on the x-axis. The relative intensity in % of the subjects’ V̇O_2peak_ that would be required to exercise at the three absolute intensities (2, 3, and 6 METs) is depicted on the y-axis. Intensity categories are marked by the dashed lines. LPA is defined as 0 to < 46% of V̇O_2peak_, MPA as 46 to < 64% of V̇O_2peak_, and VPA as 64 to 100% of V̇O_2peak_.^2^ The hatched area symbolizes the intensity that cannot be maintained for a prolonged time, as it exceeds the individual’s cardiorespiratory fitness. Abbreviations: LPA, light physical activity; MPA, moderate physical activity; VPA, vigorous physical activity; V̇O_2peak_, peak oxygen uptake.
